# An overview of genes and mutations associated with Chlamydiae species’ resistance to antibiotics

**DOI:** 10.1186/s12941-021-00465-4

**Published:** 2021-09-03

**Authors:** Ichrak Benamri, Maryame Azzouzi, Kholoud Sanak, Ahmed Moussa, Fouzia Radouani

**Affiliations:** 1 Chlamydiae and Mycoplasma Laboratory, Institut Pasteur du Maroc, 20360 Casablanca, Morocco; 2 Systems & Data Engineering Team, National School of Applied Sciences, Abdelmalek Essaadi University, Tangier, Morocco; 3 Laboratory of Microbiology, Pharmacology, Biotechnology and Environment, Faculty of Sciences Aîn-Chock, Hassan II University, Casablanca, Morocco; 4 IRDA Team, ENSIAS Mohammed V University, Rabat, Morocco

**Keywords:** Chlamydiae, Infections, Antibiotics, Resistance, Genes, Mutations, Dataset

## Abstract

**Background:**

Chlamydiae are intracellular bacteria that cause various severe diseases in humans and animals. The common treatment for chlamydia infections are antibiotics. However, when antibiotics are misused (overuse or self-medication), this may lead to resistance of a number of chlamydia species, causing a real public health problem worldwide.

**Materials and methods:**

In the present work, a comprehensive literature search was conducted in the following databases: PubMed, Google Scholar, Cochrane Library, Science direct and Web of Science. The primary purpose is to analyse a set of data describing the genes and mutations involved in Chlamydiae resistance to antibiotic mechanisms. In addition, we proceeded to a filtration process among 704 retrieved articles, then finished by focusing on 24 studies to extract data that met our requirements.

**Results:**

The present study revealed that *Chlamydia trachomatis* may develop resistance to macrolides via mutations in the *23S rRNA*, *rplD*, *rplV* genes, to rifamycins via mutations in the *rpoB* gene, to fluoroquinolones via mutations in the *gyrA*, *parC* and *ygeD* genes, to tetracyclines via mutations in the *rpoB* gene, to fosfomycin via mutations in the *murA* gene, to MDQA via mutations in the secY gene. Whereas, *Chlamydia pneumoniae* may develop resistance to rifamycins via mutations in the *rpoB* gene, to fluoroquinolones via mutations in the *gyrA* gene. Furthermore, the extracted data revealed that *Chlamydia psittaci* may develop resistance to aminoglycosides via mutations in the *16S rRNA* and *rpoB* genes, to macrolides via mutations in the *23S rRNA* gene. Moreover, *Chlamydia suis* can become resistance to tetracyclines via mutations in the *tet(C)* gene*.* In addition, *Chlamydia caviae* may develop resistance to macrolides via variations in the *23S rRNA* gene. The associated mechanisms of resistance are generally: the inhibition of bacteria’s protein synthesis, the inhibition of bacterial enzymes’ action and the inhibition of bacterial transcription process.

**Conclusion:**

This literature review revealed the existence of diverse mutations associated with resistance to antibiotics using molecular tools and targeting chlamydia species’ genes. Furthermore, these mutations were shown to be associated with different mechanisms that led to resistance. In that regards, more mutations and information can be shown by a deep investigation using the whole genome sequencing. Certainly, this can help improving to handle chlamydia infections and healthcare improvement by decreasing diseases complications and medical costs.

**Supplementary Information:**

The online version contains supplementary material available at 10.1186/s12941-021-00465-4.

## Background

Chlamydiae are Gram-negative bacteria; obligate intracellular pathogens and symbionts of diverse organisms, ranging from human to amoebae [1]. The best studied group in Chlamydiae phylum is the Chlamydiaceae family, which comprises of 11 species that are pathogenic to humans and animals [1]. *Chlamydia trachomatis* and *Chlamydia pneumoniae* represent the main human pathogenic species. They are responsible for a wide range of diseases. Indeed, *C. trachomatis* is a pathogen responsible for ocular and urogenital infections [2–4], while, *C. pneumoniae* is strongly involved in respiratory diseases and described to be associated with atherosclerosis [3, 5–8]. Whereas, *Chlamydia psittaci* can be transmitted accidently to humans, causing respiratory tract problems [9]. The mouse pathogen *Chlamydia muridarum* serves as an experimental model for genital tract infection studies [10].

Although Chlamydiae are susceptible to a wide variety of antibiotics that interfere with DNA and protein synthesis, including tetracyclines, macrolides, fluoroquinolones, rifamycins and lincosamides [11], no drug is sufficiently cost effective for the elimination of the bacterium in developing nations, and an effective vaccine has thus far been elusive [10, 12, 13].

The antibiotics misuse, for instance overuse or self-medication are known as risk factors to increase the appearance of chlamydia species resistance to antibiotics in the recent years, these factors pose a critical and serious public health problem worldwide [14]. Indeed, Chlamydia species develop resistance to antibiotics through diverse mechanisms [15], the resistance to azithromycin frequently results from mutations in the peptidyl transferase region of the *23S rRNA* genes, which impacts the reversible binding to the large ribosomal subunit near the peptidyl-transferase center and a bacteriostatic effect (bacterial growth) due to protein synthesis inhibition [16, 17]. In addition, resistance to tetracycline is often associated with foreign genomic islands integrated in chlamydial chromosome, which impacts the inhibition of bacteria protein synthesis by binding to their ribosome (with a high affinity to 30S subunit) and preventing the attachment of amino acyl-tRNA at the acceptor site [18]. Whereas, the resistance to fluoroquinolone mechanism was shown to be associated with the presence of mutation in the *gyrA* quinolone resistance determining region, which impacts the inhibition of two bacterial enzymes of the class II topoisomerase family—DNA gyrase and DNA topoisomerase IV [19]. For resistance to rifampin, a nucleotide substitution in *rpoB* gene is responsible, impacting the inhibition of bacterial transcription by interacting with beta-subunit of bacterial DNA-dependent RNA polymerase [20].

The main purpose of this study was to conduct a literature review to extract genes and mutations related to antibiotic resistance in all Chlamydia species, which will help making decisions about new research and new drugs.

## Materials and methods

**Fig. 1:**
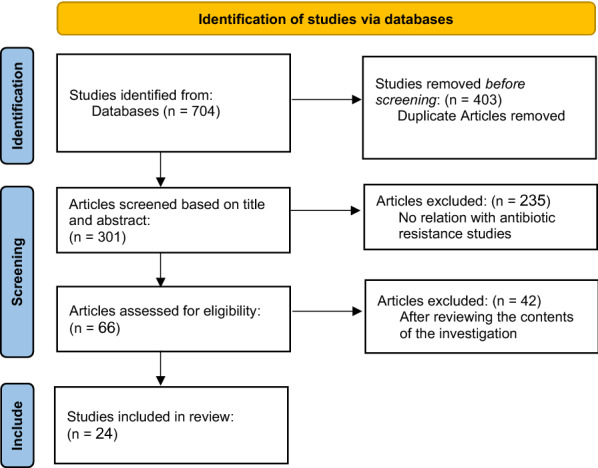
Flow chart of the literature search and study selection process

A comprehensive literature search was conducted using several databases including PubMed, Google Scholar, Cochrane Library, Sciencedirect, Web of Science. The search terms included a combination of keywords such as: Chlamydia OR Chlamydiae AND Infection OR Species OR *C. trachomatis* OR *C. pneumoniae* OR *C. psittaci* OR *C. suis* OR *C. caviae* AND Antibiotic AND Resistance AND Gene AND Mutation. The purpose of our research is to extract all the studies related to antibiotic resistance in Chlamydia species. We identified and collected all articles published after 1990. We followed a selection process (PRISMA methodology [21]) to identify the most informative studies, the flow diagram below summarizes this process (Fig. 1) [22].

Among 704 retrieved publications, 24 papers were retained as articles meeting our criteria after a meticulous two-step filtration process. The first step was the analysis of the titles and abstracts to retain relevant manuscripts. Eventually, the full texts of the manuscripts were analysed to extract information about the existing variations.

## Results and discussion

**Fig. 2:**
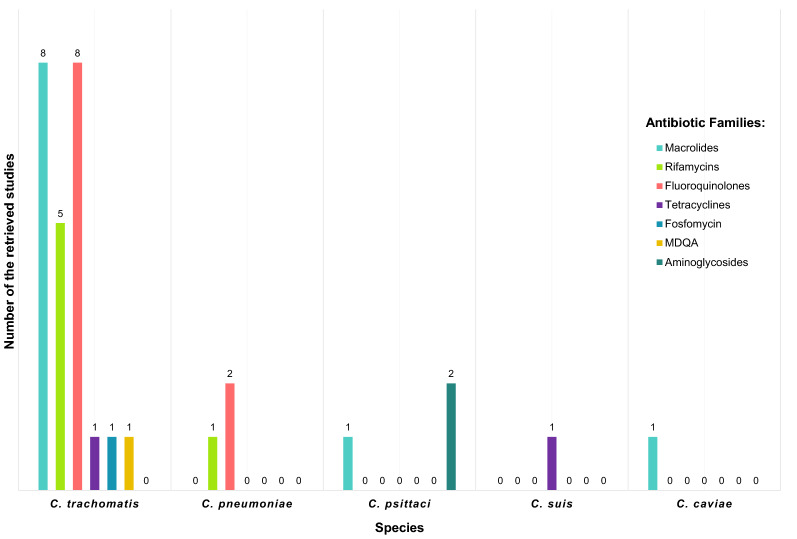
Studies sorted by Chlamydia species and antibiotic families

This literature review regrouped the total retrieved studies, which investigated the genes and mutations associated with chlamydia species’ resistance to antibiotics. The retrieved studies concerned mainly *C. trachomatis, C. psittaci*, *C. pneumoniae*, *C. suis* and *C. caviae*. The results were presented and illustrated by Chlamydia species, then sorted by antibiotic family (Fig. 2).

### Genes and mutations associated with *Chlamydia trachomatis* resistance to antibiotics

#### Resistance to macrolides

##### Mutations in the *23S rRNA* gene

The purpose of the study performed by Jiang et al. [23] was to investigate the mutations retrieved in the *23S rRNA* gene and their impact on the resistance in *C. trachomatis* clinical isolates and wild type strains to erythromycin, azithromycin and josamycin. The *C. trachomatis* isolated from patients who were treated in Tianjin Branch of STD, China from 2005 to 2008, following serial passages for 13 selected resistant mutants. The results revealed three isolates with the mutation T2611C in the *23S rRNA* gene from the eight wild type isolates resistant to erythromycin, whereas the others didn’t show mutations. In addition, the same mutation T2611C was identified in ten mutant isolates, the A2057G mutation was shown in six isolates and the A2059G in only one isolate. Furthermore, the selected mutations by exposure to subinhibitory concentrations of macrolides and the macrolide mutations selected in resistant isolates of the wild type strain were different. It was also considered that other mechanisms could be responsible for *C. trachomatis* resistance to macrolide since no mutation was identified in the *23S rRNA* gene of some isolates.

The same mutations have been found for the first time in the study performed by Zhu et al. [24], where 13 *C. trachomatis* E-UW-5/CX serotype strains were exposed to serial passage to sub-inhibitory concentrations of erythromycin (0.5 µg/ml), azithromycin (0.5 µg/ml) and josamycin (0.04 µg/ml) to select macrolide resistant mutants. The study revealed that *C. trachomatis* mutants were shown to become resistant to antibiotic at low levels of macrolides (1/2 MIC). Furthermore, the partial sequencing of the *23S rRNA* genes of susceptible and resistant *C. trachomatis* strains was performed. The mutations A2057G, A2059G and T2611C are related to the resistance of *C. trachomatis* in the peptidyl transferase region of the *23S rRNA* gene, their presence was retrieved in six mutants, two mutants and ten mutants respectively for A2057G, A2059G and T2611C.

In another study performed by Misyurina et al. [25], the objective was to explore the resistance of *C. trachomatis* clinical isolates to macrolides and investigate possible mutations in *23S rRNA* gene. The studied *C. trachomati*s isolates (n = 4) were obtained from four patients enrolled in the D.O. Ott Institute of Obstetrics and Gynaecology (Saint Petersburg, Russia) and results revealed that resistance to erythromycin, azithromycin and josamycin was associated to the two mutations A2058C and T2611C in the *23S rRNA* gene.

In another study by Xue et al. [26], they established an in vitro McCoy cell model of azithromycin induced persistent infection, for that purpose, the serovars D and F of *C. trachomatis* were used to explore the *23S rRNA* gene mutations, specifically A2057, A2058 or A2059 in the peptidyl transferase region have been mapped. The results suggested that resistance to azithromycin in a persistent state wasn’t linked to these mutations. In contrast, the studies [26–28] targeting *C. trachomatis* isolated didn’t show mutations in the *23S rRNA* genes.

##### Mutations in the *rplD* gene (encoding L4 protein)

In a study performed by Zhu et al. [24], the *rplD* gene which encodes L4 protein, 13 *C. trachomatis* strains were subjected to subinhibitory concentrations of erythromycin azithromycin and josamycin with MIC (0.5 µg/ml), (0.5 µg/ml) and (0.04 µg/ml) respectively during the cell culture serial passages to select macrolide resistant mutants. The PCR amplification and DNA sequencing showed double mutations in the L4 ribosomal protein in all mutants: Pro109(CCG) $\to$ Leu(CTG) and Pro151(CCG) $\to$ Ala(GCC) in the corresponding protein compared with the published GenBank sequence. Binet et al. [29] also explored the mutations in the *rplD* gene of *C. trachomatis* strains serovar L2/LGV/434/Bu. In this purpose, they cultivated the bacteria in mouse fibroblast L2 cells, and established the susceptibilities to azithromycin, erythromycin, josamycin, spiramycin, clindamycin, virginiamycin M1 and chloramphenicol, then PCR/DNA sequencing were performed. The results showed the one single C196A mutation leading to a Gln66 $\to$ Lys change in ribosomal protein L4 of *C. trachomatis* L2. Furthermore, the phenotypes of sensitivity associated with the mutation Q66K in the protein L4 of *C. trachomatis* L2 were accorded to an eightfold decrease in azithromycin MIC (0.8 µg/ml) and erythromycin MIC (0.8 µg/ml), in addition to a fourfold decrease in josamycin MIC (0.2 µg/ml) and spiramycin MIC (4 µg/ml). In contrast, the studies of [27, 28] targeting *C. trachomatis* isolated didn’t show mutations in the *rplD* gene.

##### Mutations in the *rplV* gene (encoding L22 protein)

Deguchi et al. [28] enrolled 7 patients showing treatment failure following the use of extended-release azithromycin and detected the mutations G52S, R65C and V77A in three DNA specimens of five examined L22. These mutations were situated outside the region closest to macrolide binding site, and related MICs of azithromycin and josamycin were (0.08 µg/ml) and (0.04 µg/ml) respectively.

The same variations (Gly52(GGC) $\to$ Ser(AGC), Arg65(CGT) $\to$ Cys(TGT), and Val77(GTC) $\to$ Ala(GCC)) were retrieved by Misyurina et al. [25] in the L22 protein of three *C. trachomatis* strains, obtained from Russian patients with urogenital infections. However, their role in *C. trachomatis* resistance wasn’t evaluated. These variations are located in a non-conserved region of the L22 protein.

#### Resistance to rifamycins

To evaluate the risk of chlamydial antibiotic resistance, Rupp et al. [30] examined the development of resistant mutant in a quantitative perspective. In that context, *Chlamydia trachomatis* serovars L2 (ATCC VR-902B) and D (ATTC VR-885) clones’ infectious elementary bodies were purified, then rifampin and moxifloxacin mutant’s resistant were detected by a plaque assay for antibiotic resistant clones. In addition, the variations were analysed in the resistant clones in different genes, mainly, the *rpoB* gene was investigated in 30 rifampicin resistant mutants of *C. trachomatis* serovar L2 and 15 rifampicin resistant mutants of *C. trachomatis* serovar D, they were subjected to culture to explore the presence of mutations and MIC determination. Firstly, the authors found rifampin resistant mutants in *C. trachomatis* serovar L2 with MIC (4–128 mg/l), different variations (n = 7) were detected at six *rpoB* loci: (Gln458 $\to$ Lys), (His471 $\to$ Asn), (His471 $\to$ Tyr), (Ala467 $\to$ Glu), (Ser476 $\to$ Leu), (Ile517 $\to$ Leu), (Gln458 $\to$ Leu). The study showed that Gln458 $\to$ Lys is the most common mutation in the serovar L2 with 15 of the 30 clones. The study also revealed rifampin resistant mutants in *C. trachomatis* serovar D with MIC (4–64 mg/l) within three different *rpoB* mutations in two loci: (His471 $\to$ Asn), (His471 $\to$ Tyr), (Ala467 $\to$ Val).

*rpoB* gene was also investigated by Suchland et al. [31], the authors explored the potential of *C. trachomatis* serovar L2 to develop resistance when it’s made under effect of rifalazil and rifampin in cell culture, the study goals were to compare the mutant selection frequency of the two antibiotics and set the MIC in mutants’ resistant. For this reason, three methods were used; the first one consisted in cloning the mutants by limiting dilution. In the second, the mutants were left as a mixed population, for both methods, serial passages were performed to reach the highest level of resistance. In the third method, the vial passage was accomplished to determine more effectively resistance to rifalazil as a selective agent. The results of method 1 and 2 showed mutants in *rpoB* for the rifampin. In a MIC (0.5 µg/ml), a single mutation I517M was found in *rpoB*, whereas for the MIC (4 µg/ml) two mutations I517M and V466A were detected, the results also showed that the highest level of resistance (512 µg/ml) was led by mutations acquisition, either D461N or H471N, in combination with I517M. For the rifalazil, the results of method 3 showed the H471N mutation in MIC (0.016 µg/ml), whereas the H471L and H471Y mutations were detected for the MIC (0.032 µg/ml), mutants with numerous mutations were retrieved H471N with I517M and H471L with I517M, on the other hand, the use of rifampin revealed multiple mutations in the *rpoB* gene (H471N with I517M, V466A with I517M, and the triple mutant V466A, A467T, and I517M).

The H471Y mutation was also detected by Kutlin et al. [32] in the study where they explored the development in vitro of phenotypic and genotypic resistance, resulting from serial passages of *C. trachomatis* (BU-434/L2 and UW-3/Cx/D) in subinhibitory concentrations of rifalazil and rifampin, they also assessed the acquisition of resistance during the exposition to the antibiotics. After six passages, *C. trachomatis* showed resistance with higher level resistance to rifampin (128–256 µg/ml) and lower-level resistance to rifalazil (0.5–1 µg/ml). In addition, PCR/sequencing revealed the mutation His471 $\to$ Tyr (H471Y) in *C. trachomatis* UW-3/Cx/D strains resistant to both antibiotics. However, Val136 $\to$ Phe (V136F) mutation was detected by sequencing of the entire *rpoB* gene of *C. trachomatis* BU-434/L2 resistant to rifalazil, whereas, no mutation was retrieved in the *rpoB* gene of *C. trachomatis* BU-434/L2 highly resistant to rifampin.

In another study, Binet et al. [33] investigated in the sensitive *Chlamydiaceae* strains the frequencies of spontaneous resistance to spectinomycin and rifampin. *C. trachomatis* serovar L2/LGV/434/Bu strains were processed by culture in fibroblast L2 cells of mouse. The authors sequenced the *rpoB* gene to detect the changes associated with rifampin resistance, the results showed a single base pair substitution in the *rpoB* sequences of the rifampin resistant variant, creating an allele GAU $\to$ GGU (Asp516 $\to$ Gly). This mutation conferred a high level of rifampin resistance with MIC (800 ng/ml), and a frequency of resistance (i.e., 10^–7^).

In another study, Dresses-Werringloer et al. [34] established an in vitro cell culture model in epithelial cells infected by *C. trachomatis* serovar K to study the long-term effect of rifampicin. In that purpose, a 656 bp fragment from the *rpoB* gene variants was sequenced from five *C. trachomatis serovar* K resistant to rifampicin, selected in vitro from a wild type parent. The sequence analysis revealed the three variants Ala467 $\to$ Val with MIC (4 µg/ml), two variants His471 $\to$ Tyr for high level resistance MICs (64–256 µg/ml) with the following additional genetic variation.

#### Resistance to fluoroquinolones

##### Mutations in the *gyrA*

This literature review also revealed a number of mutations associated with resistance to fluoroquinolone. Deguchi et al. [28] examined the presence of mutations associated to fluoroquinolone in the *gyrA* gene in *C. trachomatis* isolated from the first void urine samples of men suffering from acute urethritis. The quinolone resistance determining region (QRDR) of the *gyrA* gene was analysed in 118 specimens, the sequenced region of the *gyrA* in 44 DNA specimens showed V61A and H129Q variations, these mutations were not localized in the QRDR of the *gyrA* gene. The same mutation V61A has been found by Yokoi et al. [35] in *gyrA* gene in seven *C. trachomatis* clinical isolates from men with chlamydial nongonococcal urethritis (NGU). They amplified the *gyrA* gene QRDR and the *parC* gene analogous region of *C. trachomatis* clinical strains, then examined the presences of mutations associated with the resistance to fluoroquinolone. For this reason, only six *C. trachomatis* isolates have been examined to determine the antibiotic susceptibilities, and one presented the substitution Cys66 $\to$ Arg in *gyrA* gene.

To explore the resistance of *C. trachomatis* serovar L2 strains to fluoroquinolones, Dessus-Babus et al. [36] and Geisler et al. [37] exposed *C. trachomatis* L2 to fluoroquinolones serially in vitro and selected mutants with fluoroquinolone resistance. The results showed the substitution Ser83 $\to$ Ile (G $\to$ T) in the *gyrA* gene, this mutation was also detected in various bacterial species.

In another hand, Rupp et al. [30] explored the development of resistant mutant in a quantitative perspective for *Chlamydia trachomatis* serovars L2 (ATCC VR-902B) and D (ATTC VR-885). They established a new in vitro test to select the mutation frequency in the QRDR of *gyrA*. The results showed transversions Ser83 $\to$ Ile or Ser83 $\to$ Arg in the in the *gyrA* gene in almost identical MICs of (16–32 mg/l). Furthermore, Morrissey et al. [38] examined the resistance of *C. trachomatis* to fluoroquinolone and results revealed the substitution Ser83 $\to$ Iso (AGT $\to$ ATT) in *gyrA* QRDR of both *C. trachomatis* mutant strains.

The *gyrA* was also explored by Shkarupeta et al. [39] and sensitivity to fluoroquinolone was studied in vitro by cell culture. Furthermore, the mutations associated to resistance to fluoroquinolone in *gyrA* gene of *C. trachomati*s clinical strains, isolated after treatment failure from cervical samples of women and urethral samples of men. The authors reported that some isolates presented the mutations Val60 $\to$ Ala (GTA $\to$ GCA) and His129 $\to$ Gln (GAC $\to$ GAG) in the QRDR neighbouring *gyrA* region. In addition, the 3D model of *C. trachomatis* DNA-gyrase subunit structure exhibited remote spatial location of amino acid residues 60 and 129 compared to the location of the familiar “hot points”. These variations were only retrieved in *C. trachomatis* serovar E clinical isolates with different MICs for levofloxacin and ofloxacin; these types of mutations weren’t detected in genotypes H and G isolates. The authors suggested that retrieved substitutions are specific to *gyrA* gene polymorphism of genotype E. In the other hand, Misiurina et al. [40] reported that three *C. trachomatis* clinical isolates resistant to fluoroquinolone with MIC ( ≥64 µg/ml) of ofloxacin didn’t present mutations in the QRDRs of the *gyrA* gene.

##### Mutations in the *parC* gene

Concerning the topoisomerase IV subunit C (*parC*) gene, Yokoi et al. [35] examined also fluoroquinolone susceptibilities for six *C. trachomatis* clinical isolates, the total examined isolates had the Arg83 $\to$ Gly substitution in *parC*. However, the studies performed by: Deguchi et al. [28] in 284 *C. trachomatis* men’s first void urine DNA positive specimens, Rupp et al. [30] in 14 *C. trachomatis* clinical isolates, and Misiurina et al. [40] in *C. trachomatis* serovars L2 and D didn’t show mutations associated to fluoroquinolones resistance in the *parC* gene.

##### Mutations in the *ygeD* gene

In addition to the studies performed in *gyrA* and *parC*, Misiurina et al. [40] found silent variations leading to amino acid substitutions in the *ygeD* gene 3' region of two isolates resistant to high and intermediate resistance levels to fluoroquinolones.

#### Resistance to tetracyclines

For resistance to tetracyclines antibiotics family, O’Neill et al. [41] focused on two *C. trachomatis* strains isolated in the USA. The isolate IU888, originally cultured from a 19 years old woman 10 months after erythromycin treatment, and originally found to be resistant to concentrations of tetracycline (64 µg/ml), the isolate IU824, cultured from the endometrium of a 27 years old woman 5 months after tetracycline and 11 months after cephalosporin treatment. The authors found that IU824 was resistant to a lower tetracycline concentration (16 µg/ml) than IU888 isolate. but IU824 and IU888 were equally resistant to the other tested antibiotics. The whole genome sequencing of IU824 and IU888 from E/Bour using the Illumina Hiseq platform allowed the detection of a unique SNP in the *ompB/porB* gene of IU824 but not IU888. The SNP resulted in a premature stop codon at codon 81, this base change from C826442T relative to the E/Bour genome verified by capillary sequencing of *porB*.

#### Resistance to fosfomycin

The fosfomycin was also targeted to study *C. trachomatis* resistance, for that, McCoy et al. [42] used genetics-based methods to reassess chlamydial abnormalities, they characterized *C. trachomatis* serovar L2 *murA* gene (UDP-*N*-acetylglucosamine enolpyruvyl transferase that catalyses the first committed step of peptidoglycan synthesis). For this objective, the authors cloned the gene from *C. trachomatis* serovar L2 to determine if chlamydial *murA* encoded a functional enzyme, a PCR was performed and amplicon was cloned into pBAD18 to place the gene under the arabinose-inducible, glucose-repressible *ara* promoter, the resulting clone pAJM6 was sequenced to confirm that the insert contained the *C. trachomatis murA*. The results showed that *C. trachomatis* L2 *murA* gene presents the mutation C119D (cysteine $\to$ aspartate), retrieved in the active site of the enzyme and *C. trachomatis* was resistant to high levels of fosfomycin.

#### Resistance to (3-methoxyphenyl)-(4,4,7-trimethyl-4,5-dihydro-1H-[1, 2] dithiolo[3,4-C]quinolin-1-ylidene) amine (MDQA)

To identify novel compounds that inhibit chlamydial growth in mammalian cells, Sandoz et al. [43] processed to a serial passage to generate a completely resistant mutant by increasing concentrations of (3-methoxyphenyl)-(4,4,7-trimethyl-4,5-dihydro-1H-[1, 2] dithiolo[3,4-C]quinolin-1-ylidene) amine (MDQA) until passing the wild type parental strains MIC. The results concentration curve showed that the mutant strain and the wild type strains grows equally in the presence and the absence of MDQA, and the mutations Ala420 $\to$ Pro, Ala246 $\to$ Pro and Ala45 $\to$ Ser in the *secY* were found in the three resistant strains, these variations map to the *secY* translocon central channel and can modify the channel structure.

### Genes and mutations associated with *Chlamydia pneumoniae* resistance to antibiotics

#### Resistance to rifamycins

To assess the potential establishment of resistance during treatment, Kutlin et al. [32] investigated in vitro the effect of serial passage of *C. pneumoniae* (TW-183 and CDC/CWL-029) in subinhibitory concentrations of rifalazil and rifampin on the phenotypic and genotypic resistance apparition. The results showed that after twelve passage, *C. pneumoniae* (TW-183) acquired low level resistance to rifampin and rifalazil with MICs (0.25 µg/ml) and (0.016 µg/ml) respectively, at the genomic level, two mutations were selected in the predicted rifamycin resistance region of *C. pneumoniae* TW-183 *rpoB* gene: the Leu456 $\to$ Iso (L456I) associated to resistance to rifampin and the Asp461 $\to$ Glu (D461E) associated to resistance to rifalazil. However, *C. pneumoniae* CWL-029 didn’t acquire resistance to the two antibiotics.

#### Resistance to fluoroquinolones

The resistance to fluoroquinolone of *C. pneumoniae* CV-6 coronary artery isolate, obtained from a male healed from a chronic atheromatous infection injury was explored by Rupp et al. [44]. They used serial passage and performed subcultures of *C. pneumoniae* CV-6 with increase of moxifloxacin concentrations (0.0125–6.4 mg/l), this resulted to an increase of 256-fold MIC compared to moxifloxacin naive strains with a novel point mutation Ser $\to$ Asn (G83A). This variation initiated the recognition site 5′-A^*▼*^CGT-3′ (cutting site for the enzyme of restriction endonuclease HpyCH4IV).

In another hand, Morrissey et al. [38] studied the phenotypic resistance of *C. pneumoniae* IOL 207 strain to ofloxacin, sparfloxacin and moxifloxacin using serial passage on McCoy cell monolayers. The results revealed that *C. pneumoniae* type strain IOL 207 didn’t show resistance to moxifloxacin and ofloxacin during the passages. In addition, the resistance to sparfloxacin needs further investigation to show the possibility of emergence.

### Genes and mutations associated with *Chlamydia psittaci* resistance to antibiotics

#### Resistance to aminoglycosides

##### Mutations in the *16S rRNA* gene

Conversely to the previous studies, Binet et al. [45] interested to the fitness costs of spectinomycin resistance in *C. psittaci* 6BC to determine the frequency in vivo of a resistance determinant, they induced mutations and identified the phenotypic changes that occur from mutations in the *16S rRNA* gene related to spectinomycin resistance of *C. psittaci* 6BC. For this purpose, the authors studied the growth of four genetically induced clonal variants in *C. psittaci* 6BC resistance to spectinomycin**,** where three growth patterns were observed: the first pattern was a development of *C. psittaci* 6BC 16S1 (BC0E1) and 16S3 (BCS18) resulting to the mutations C1192U and C1192G respectively, this growth was identical to the parental strain except an initial delay in DNA replication, inducing a resistance to spectinomycin with MIC (> 10 µg/ml). The second pattern was the growth of *C. psittaci* 6BC 16S4 (BCS34) resulting to the mutation C1193G, with a delay in the production of EBs, inducing resistance to spectinomycin with MIC (5 µg/ml). The third pattern was the growth of *C. psittaci* 6BC 16S2 (BC0A2) resulting to the mutation A1191G, which showed the formation of RBs and EBs during the developmental cycle slower than the normal, inducing a resistance to spectinomycin with MIC (> 10 µg/ml).

In another study, Binet et al. [33] investigated the frequency of resistance to spectinomycin in 53 *C. psittaci* 6BC spectinomycin resistant variants, which were purified from individual spectinomycin resistant strains grown in the presence of the antibiotic, the spontaneous spectinomycin resistant mutants appeared with a frequency of (5 × 10^–5^). The results showed 46 mutants presenting the same mutation C1192U like that found in *C. psittaci* 6BC 16S1 (BC0E1) with MIC (> 10 mg/ml) and five mutants presenting the same mutation A1191G like that found in *C. psittaci* 6BC 16S2 (BC0A2) with MIC (> 10 mg/ml). Moreover, the mutation C1192G in *C. psittaci* 6BC 16S3 (BCS18) and the mutation G1193C in *C. psittaci* 6BC 16S4 (BCS34) were detected in high level of spectinomycin with MICs (> 10 mg/ml) and (5 mg/ml) respectively.

##### Mutations in the *rpoB* gene

In the same study, for the same objective following the same protocol, Binet et al. [33] explored the frequency of resistance to rifampin in *C. psittaci* 6BC. Indeed, degenerate primers were used to amplify both *rpoB* regions from *C. psittaci* 6BC rifampin resistant (BCR1) and wild type chlamydial parent, then, *rpoB* gene was sequenced. The results showed a single base pair substitution in the *rpoB* gene sequences of the rifampin resistant variant creating an allele AUG $\to$ AUC (Met515 $\to$ Ile) (designed *rpoB*2 for BCR1), this mutation conferred a high level of rifampin resistance with MIC (300 ng/ml), with a frequency of (i.e., 10^–7^).

#### Resistance to macrolides

In the same goal as for *C. trachomatis*, Binet et al. [29] examined the contribution in vitro of spontaneous variation in the chromosome associated with emergence of resistance of *C. psittaci* to azithromycin. In that objective, *C. psittaci* serovar 6BC was cultivated in mouse fibroblast L2 cells, and susceptibility to azithromycin, erythromycin, josamycin, spiramycin, clindamycin, virginiamycin M1, and chloramphenicol were determined. In a second step, the PCR/DNA sequencing was used to decide if the azithromycin resistance resulted from variation in *23S rRNA*, *rplD* or *rplV* genes. On the other hand, a coinfection at a ratio of 1:1 of *C. psittaci* 6BC (BC_RB_) wild type and an isogenic representative of each azithromycin resistant variant was performed. In addition, to determine the sequence of azithromycin binding site in the *23S rRNA* gene, a 1400 bp PCR fragment amplified from *C. psittaci 6BC* parent strain and 30 azithromycin resistant mutants were sequenced. The results revealed only one mutation among the following A2058C, A2059C, or A2059G in the *23S rRNA* gene of each studied mutant. In addition, the authors selected one azithromycin resistance representative of each mutant category from the 6BC clone population of *C. psittaci*, namely BC_RB_AZ1, BC_RB_AZ2 and BC_RB_AZ5, and extended the two development cycles for more phenotypic and physiological characterization on the bacterial population. The results didn’t reveal changes in the DNA sequence of the *rplD* and *rplV* genes. In contrast to resistance to azithromycin, *C. psittaci* 6BC was found to be sensitive to the 14-member-ring erythromycin MIC (200 ng/ml), the 15-member-ring azalide azithromycin MIC (100 ng/ml), the 16-member-ring josamycin MIC (50 ng/ml) and spiramycin MIC (1 g/ml). And growth inhibition of *C. psittaci* 6BC by both clindamycin and lincosamide was observed with MIC (400 ng/ml), however the growth inhibition by virginiamycin M1 and streptogramin was with MIC (2 g/ml). Moreover, the authors analysed the biological costs linked to mutations in the *23S rRNA* gene associated with *C. psittaci* resistance to macrolide, for that, they compared the growth of the susceptible parent to that of the isogenic macrolide resistant variants in the absence of selection. It seems from the prolonged eclipse of the development cycle that infectious particles formation for each mutant was delayed, moreover, each mutant was highly surpassed by the wild type strain at the end of the cycle.

### Genes and mutations associated with *Chlamydia suis* resistance to antibiotics

In their study, Dugan et al. [46] investigated *C. suis* resistance to tetracycline to elucidate the mechanism related to this resistance. The authors enrolled *C. suis* resistant strains to tetracycline, isolated from pigs (R19, R24, R27, H5, H7, 130 and 132) and *C. suis* sensitive strain (S45) to tetracycline. They used Vero cells culture in 96-well plates and performed a tetracycline twofold serial dilution (0.3–40 µg/ml) to determine each *C. suis* strain MICs. For DNA amplification, they used specific primers for 13 different tetracycline resistance genes *(tet(A), tet(B), tet(C), tet(D), tet(E), tet(G), tet(H), tet(K), tet(L), tet(M), tet(O), tet(Q),* and *tet(S)).* The genomic analysis demonstrated that the *tet(C)* was present in the all the studied resistant strains, and sequencing demonstrated a high degree of similarity between the *tet(C)* islands and the plasmid pRAS3.2, the sequences shared include all regions of each *tet(C)* island with the exception of the IScs605 element. The genomic islands are horizontally acquired DNA integrated into chlamydiae natural isolates.

### Genes and mutations associated with *Chlamydia caviae* resistance to antibiotics

The resistance of *C. caviae* to antibiotics was explored by Binet et al. [47], they analysed the biological costs of the *23S rRNA* mutations leading to *C. caviae* resistant to azithromycin by cell culture and in vivo in its natural host. In that objective, *C. caviae* serovar 6BC was cultivated in mouse fibroblast L2 cells, and susceptibility to azithromycin was determined. The DNA analysis of spontaneous azithromycin resistant showed one mutation A2058C, A2058G, A2059C or A2059G in the *23S rRNA* gene in each resistant mutant. Furthermore, two independent azithromycin resistant mutants (SP6AZ_1_ with A2058C and SP6AZ_2_ with A2059C) were compared for growth in pure culture or in competition with their isogenic parent (SP_6_), in vitro and in vivo. The results of the growth of *C. caviae *in vitro revealed that cost of azithromycin resistant mutations in *23S rRNA* gene conferred resistance to azithromycin with MIC (> 20 µg/ml), resulting to a delay of transition from RB to EB. On the other hand, the authors infected both eyes conjunctiva of guinea pig by *C. caviae* resistant mutants, the bacteria mutations A2058C and A2059C in the *23S rRNA* gene had no effect on the infection capacity and its growth in the natural environment. Moreover, when wild type and mutant *C. caviae* were mixed and inoculated in the conjunctiva of guinea pigs, the competition indices revealed that azithromycin mutant were outcompeted by the azithromycin sensitive parent strain in the ocular infection.

**Fig. 3:**
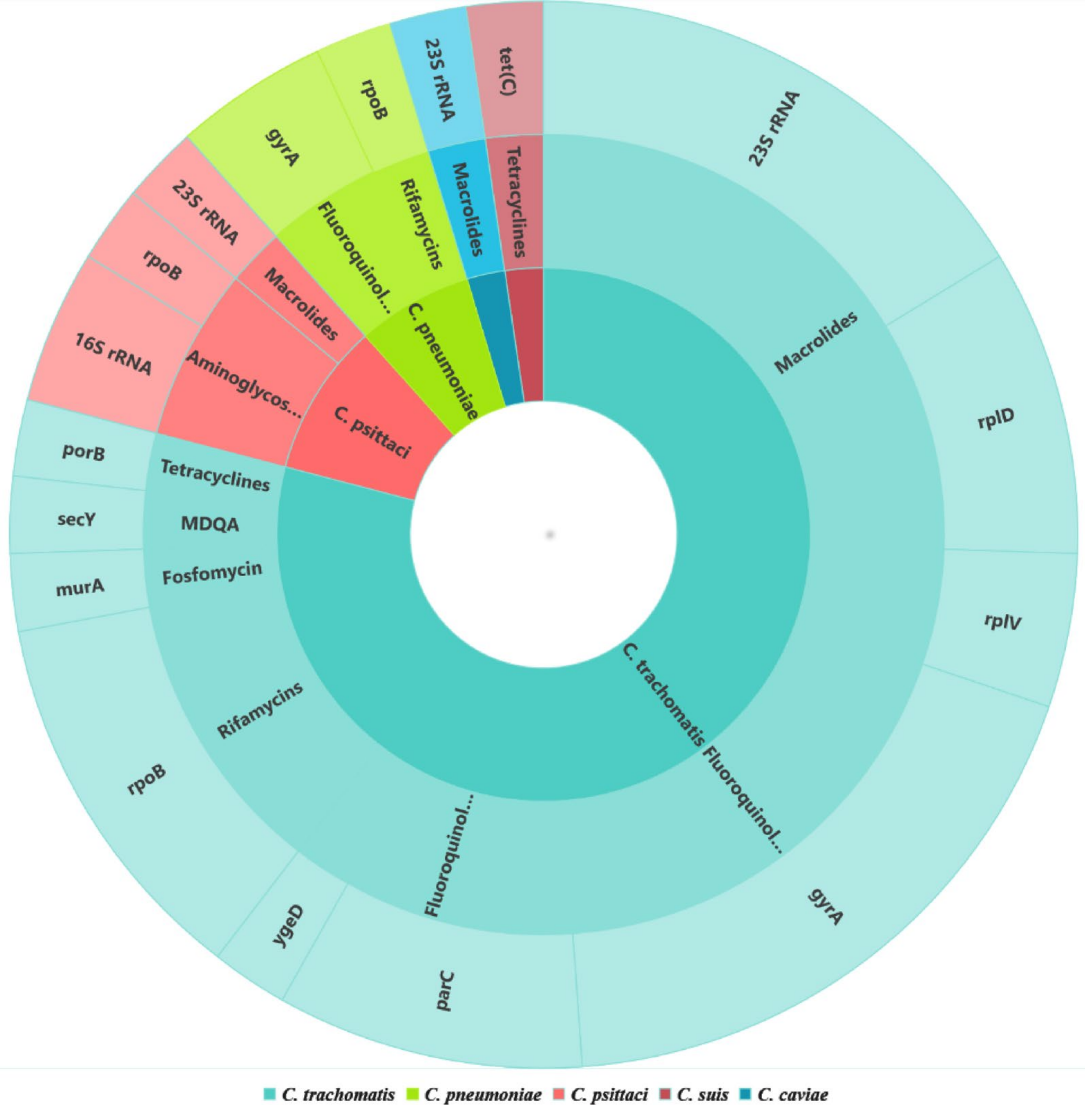
Genes involved in antibiotic resistance for Chlamydia species

In summary, genes associated with chlamydia resistance to antibiotics in both humans and animals are summarised in Fig. 3. These genes are mainly: *23S rRNA* (macrolides), *rplD* (macrolides), *rplV* (macrolides), *rpoB* (aminoglycosides and rifamycins), *gyrA* (fluoroquinolones), *parC* (fluoroquinolones), *ygeD* (fluoroquinolones), *porB* (tetracyclines), *murA* (fosfomycin), *secY* (MDQA), *16S rRNA* (aminoglycosides) and *tet(C)* (tetracyclines). These genes carried different mutations in various locations. This literature review also revealed that most of the detected mutations are located in the *23S rRNA* gene, furthermore, the mutations were shown to be associated with different mechanisms that led to resistance to antibiotics in different chlamydiae strains. Certainly, these mechanisms should have their impacts not only on the encoded proteins’ structures, characteristics but also on their functionalities. We suggest here to perform more and deep investigations to explore and better understand antibiotic resistance concerns that impact both human and animal health.

Subsequently, the extracted information (Reference Number, Author Name, Study Year, Species, Antibiotics, Genes, Mutations, Sequencing Method) from the retained studies has been provided as supplementary material (see Additional file 1).

## Conclusions

The present literature review revealed the adoption of different methodologies to conduct the genetic studies to detect and identify the genes and mutations associated with chlamydiae resistance to antibiotics. Among others, we can cite the RFLP, the PCR sequencing, the genomic islands detection which allowed the resistance mechanisms comprehension and the exploration of some associated physiological costs and fitness cost due to the associated mutations.

However, more needs to be explored. For this, we recommend the whole genome sequencing (NGS) as new technology for genomic variations exploration. With these techniques we will be enabled to have big coverage and increase the screening levels to detect variations associated with resistance to antibiotics. Certainly, this will broaden the horizon to understand better antibiotic resistance mechanisms and adopt appropriate and personalized drugs, why not to develop new drugs as a solution to these public health problems.

We also understand through this literature review some of the antibiotic resistance origins, concerns and some of their consequences, we explored some of the results that can be led to the resistance. In addition, this study can persuade for deep investigations, such investigations may allow public health workers to better explore antibiotic resistance in different chlamydia species, it also will help investigators and physicians to improve the healthcare quality and infected patient’s therapeutic surveillance, to decrease the longer hospital stays, the higher medical costs and decrease the diseases complications.

## Supplementary Information

**Additional file 1.** Details of the extracted data from the retrieved studies.

## Data Availability

Not applicable.

## Abbreviations

*C. trachomatis*: *Chlamydia trachomatis*
*C. pneumoniae*: *Chlamydia pneumoniae*
*C. psittaci*: *Chlamydia psittaci*
*C. muridarum*: *Chlamydia muridarum*
*C. suis*: *Chlamydia suis*
*C. caviae*: *Chlamydia caviae*
DNA: Deoxyribonucleic acid
MIC: Minimal inhibitory concentration
*E. coli*: *Escherichia coli*
PCR: Polymerase chain reaction
NGS: Next generation sequencing
QRDR: Quinolone resistance determining region
NGU: Chlamydial nongonococcal urethritis
SNP: Single nucleotide polymorphism
MDQA: (3-Methoxyphenyl)-(4,4,7-trimethyl-4,5-dihydro-1H-[1, 2] dithiolo[3,4-C]quinolin-1-ylidene) amine
EBs: Elementary bodies
RBs: Reticulate bodies
RFLP: Restriction fragment length polymorphism
